# A Case of Polyarteritis Nodosa Associated with Vertebral Artery Vasculitis Treated Successfully with Tocilizumab and Cyclophosphamide

**DOI:** 10.1155/2016/7987081

**Published:** 2016-01-20

**Authors:** Kae Watanabe, Dhanashree A. Rajderkar, Renee F. Modica

**Affiliations:** ^1^Department of Pediatrics, University of Florida, 1600 SW Archer Road, Gainesville, FL 32608, USA; ^2^Department of Radiology, University of Florida, Gainesville, FL 32608, USA; ^3^Department of Pediatric Immunology, Rheumatology and Infectious Disease, University of Florida, Gainesville, FL 32608, USA

## Abstract

Pediatric polyarteritis nodosa is rare systemic necrotizing arteritis involving small- and medium-sized muscular arteries characterized by aneurysmal dilatations involving the vessel wall. Aneurysms associated with polyarteritis nodosa are common in visceral arteries; however intracranial aneurysms have also been reported and can be associated with central nervous system symptoms, significant morbidity, and mortality. To our knowledge extracranial involvement of the vertebral arteries has not been reported but has the potential to be deleterious due to fact that they supply the central nervous system vasculature. We present a case of a 3-year-old Haitian boy with polyarteritis nodosa that presented with extracranial vessel involvement of his vertebral arteries. After thorough diagnostic imaging, including a bone scan, ultrasound, Magnetic Resonance Imaging/Angiography, and Computed Tomography Angiography, he was noted to have vertebral artery vasculitis, periostitis, subacute epididymoorchitis, arthritis, and myositis. He met diagnostic criteria for polyarteritis nodosa and was treated with cyclophosphamide, methylprednisolone, and tocilizumab, which resulted in improvement of his inflammatory markers, radiographic findings, and physical symptoms after treatment. To the authors' knowledge, this is the first report of vertebral artery vasculitis in polyarteritis nodosa as well as successful treatment of the condition using the combination cyclophosphamide and tocilizumab for this condition.

## 1. Background

Polyarteritis nodosa (PAN), also known as Infantile Kawasaki's Disease (KD), is small- to medium-sized vasculitis affecting multiple organ systems throughout the body. It accounts for 3% of childhood vasculitis in the United States and, in the pediatric age group, the onset peaks at 9 years old [1]. The disease varies in its presentation from a relatively benign cutaneous form, which may resolve with minimal treatment, to a severe systemic form that can be associated with high morbidity and mortality. Initial presentation is nonspecific and may include fever, malaise, weight loss, myalgias, and arthralgias. It most frequently affects the vasculature of the skin, muscles, kidneys, and gastrointestinal tract [2–4]. Depending on the vasculature that is involved, the patient may present with hypertension, ischemic heart disease, testicular pain, abdominal pain, hematuria, or proteinuria [2–4]. However, intracranial involvement, although rare, has also been reported and can be associated with hemorrhage or stroke if ischemia or rupture occurs [5, 6]. Extracranial involvement, to our knowledge, has not been reported, but if it occurred it could have the potential to be deleterious leading to stroke or ischemic events. Laboratory markers of systemic inflammation are usually elevated but are nonspecific of the disease [2]. Diagnosis is made according to The European League Against Rheumatism (EULAR)/Pediatric Rheumatology European Society (PReS) or American College of Rheumatology (ACR) classification criteria for childhood PAN [7]. The major diagnostic factor is evidence of vasculitis either by angiography and/or biopsy. In a large series of adult patients with PAN, combined muscle and nerve biopsies in symptomatic patients provided histologic confirmation of vasculitis in 83% whereas isolated muscle biopsies demonstrated vascular inflammation in 65%. However, valuable diagnostic imaging modalities include selective Computed Tomography (CT), Magnetic Resonance Imaging (MRI), or conventional angiography, which may demonstrate findings consistent with medium or large vessel vasculitis without biopsy [8]. Bone scans have been used as a frequent modality in evaluating extremity pain in adult patients with PAN, which has detected periosteal inflammation even prior to periosteal reactions of the bone [9, 10].

Our case is a unique presentation of PAN, including symptoms of neck pain related to vertebral artery vasculitis and cervical myositis, which to our knowledge is the first case described in pediatric PAN. Evaluation with bone scans allowed for a noninvasive means of confirmation of periosteal inflammation, detection of clinically silent periosteal involvement, and resolution after treatment. The concomitant use of intravenous (IV) steroids, cyclophosphamide with tocilizumab, was a safe and effective treatment option for this case; however its usage needs further investigation in the pediatric PAN population [11].

## 2. Case Presentation

A 3-year-old Haitian boy presented to pediatric rheumatology clinic due to persistently elevated inflammatory markers for 9 months after treatment for suspected atypical KD. His past medical history is otherwise negative other than the initial diagnosis of atypical KD. He was born and raised in the United States without travel prior to his illness. He has neither developmental issues nor significant family history of rheumatic disease. The patient was diagnosed and treated for atypical KD at an outside hospital based upon initial presentation with tactile fevers of unclear duration, dry cracked lips, extremity changes, elevated inflammatory markers including C-reactive protein (CRP) (200–300 mg/L), Erythrocyte Sedimentation Rate (ESR) (>100 mm/h), and very mild ectasia of his coronary arteries without perivascular or vessel wall edema on his initial echocardiogram. He did lack other typical findings of KD including conjunctival injection, exanthema, cervical adenopathy, and thrombocytosis. At the time of presentation his pertinent laboratories showed CRP 219 mg/L, sodium 131 mmol/L, aspartate aminotransferase (AST)/alanine aminotransferase (ALT) 44/19 IU/L, albumin 2.5 g/dL, ferritin 190 mg/mL, white blood cell (WBC) 10(3)/*μ*L, and platelet count 180 10(3)/*μ*L. He also did present with hypochromic microcytic anemia (hemoglobin (Hgb) 5.6 g/dL) that required IV iron therapy and subsequently oral iron supplementation. The decision to treat for atypical KD was made due to his mild coronary artery ectasia, history of fevers, dry cracking lips, and extremity changes in spite of lack of thrombocytosis which is a common finding. His treatment included one dose of intravenous immunoglobulin (2 g/kg/dose) and low dose aspirin therapy (3–5 mg/kg/day). He was followed at cardiology clinic three months later and had a normal cardiac evaluation including normal echocardiogram without evidence of coronary artery ectasia, perivascular brightness, or abnormal tapering. He was noted to have normal ventricular function and no pericardial effusion was noted. However, he continued to report intermittent tactile fevers without source and his laboratory evaluation showed persistently elevated CRP levels at 90 mg/L. His anemia improved (Hgb 9.8 g/dL) after oral iron therapy. Due to his persistent anemia, unclear diagnosis, and persistent inflammatory markers, he had a bone marrow biopsy and aspiration as well as hemoglobin electrophoresis that were normal. Six months after the initial presentation he developed epididymoorchitis, which was unresponsive to 4 weeks of oral antibiotic treatment with cephalexin. He continued to have persistently elevated ESR in the 90–100 (mm/hr) range at this duration, with CRP fluctuating between 51 and 90 mg/L. At their six-month cardiology appointment, the family reported that he was progressively more fatigued, developed a stiff neck, was shuffling gait, and was less active. He continued to have intermittent weekly subjective fevers. His outpatient evaluation by pediatric infectious diseases did not reveal a source of his fevers including negative blood cultures, T-spot TB test, treponemal Ab, rapid plasma reagin (RPR),* Cytomegalovirus*- (CMV-) polymerase chain reaction (PCR), Human Immunodeficiency Virus (HIV) Ab,* Brucella* Ag, hepatitis A, B, and C panel, and galactomannan. He was referred to pediatric rheumatology department for further evaluation 9 months after his initial presentation, diagnosis, and treatment for presumptive atypical KD.

**Figure 1:**
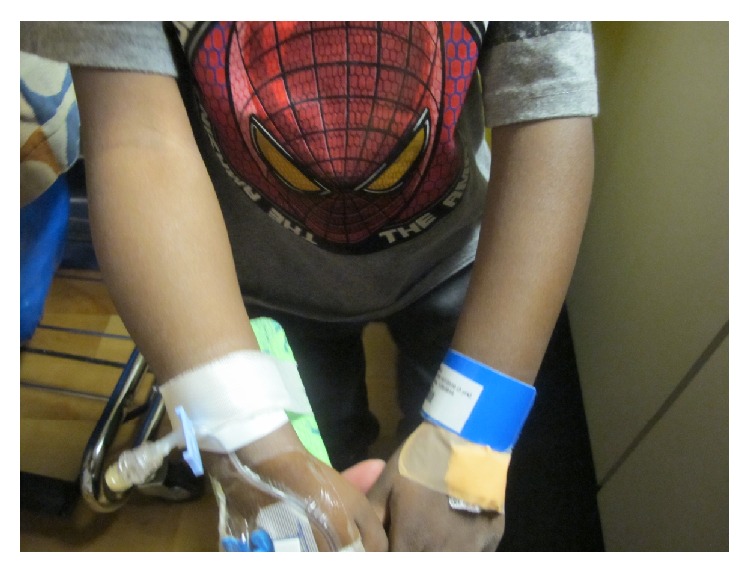
Comparison of right and left arm on physical exam. Right forearm notably enlarged compared to left. On palpation, bony enlargement and pain present circumferentially on right.

**Figure 2:**
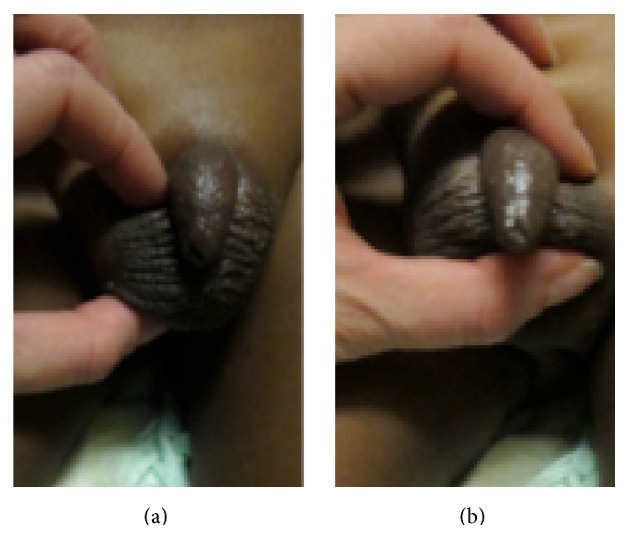
Comparison of left and right testicle on physical exam. Tender and warm right testis (a), approximately twice enlarged compared to left (b).

At his initial pediatric rheumatology appointment, the patient's physical exam showed mild torticollis with preferential tilting of his head to the right, decreased and painful extension, flexion, and lateral rotation of his cervical spine. He also was noted to have tenderness, warmth, and bony hypertrophy involving the right forearm (Figure 1) but sparing the elbow joint with normal flexion, extension, supination, and pronation of the right elbow joint. He did not have peripheral arthritis on exam and his hip rotation was normal without pain. His gait was wide based and characterized by slight hip flexion, which was thought to be related to right epididymoorchitis. His right scrotum was enlarged to about 4 cm in length with tenderness, warmth, and erythema (Figure 2).

**Figure 3:**
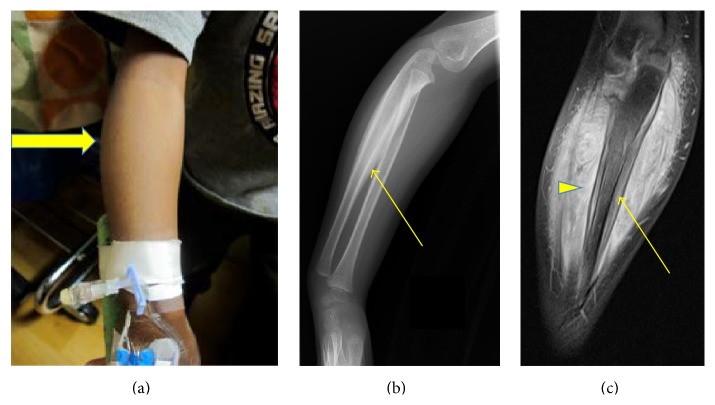
Physical findings and imaging of right arm at diagnosis. (a) Right forearm hypertrophy (yellow arrow). (b) Plain (lateral) radiograph of the right upper extremity revealing diffuse periosteal thickening of both the bones of the forearm (thin yellow arrow) without any focal lytic or sclerotic lesions. The extent and the degree of the involvement of the right ulna were marked as compared to the radius. (c) MRI T2 weighted images of the right forearm demonstrated marked diffuse, circumferential periosteal thickening involving the radius and ulna. Ulnar involvement (thin yellow arrow) was severe as compared with the radius. No focal osseous lesion was identified. Also notable was marked soft tissue involvement including the muscular compartment consistent with myositis (arrowhead).

**Figure 4:**
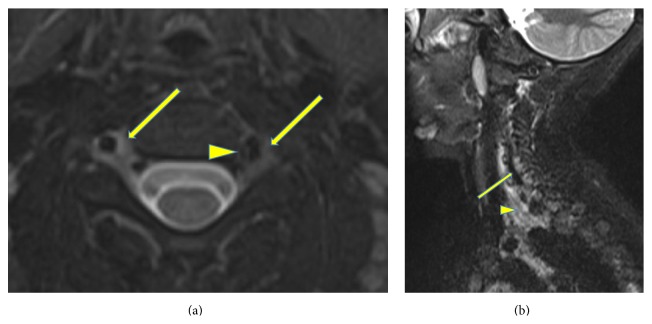
Imaging of cervical area at diagnosis. (a) Baseline MRI T2 weighted images showed moderate adventitial thickening of both the vertebral arteries at the level of C3 (thin yellow arrows) with adjacent soft tissue edema. There were focal areas of irregularity causing moderate stenosis on the left side (arrowhead). (b) Sagittal view of cervical MRI demonstrated poststenotic dilation of his left vertebral artery (arrow) and marked myositis (arrowhead).

X-rays and MRI of right forearm, MRI of neck, and Magnetic Resonance Angiography (MRA) of neck, chest and abdomen, and pelvis were recommended to evaluate ostitis, tenosynovitis, myositis, and systemic vasculitis, respectively. The right forearm X-ray showed periosteal thickening and right forearm MRI showed diffuse smooth circumferential periosteal reaction and thickening and enhancement of right ulna, as well as myositis (Figure 3). MRI of the neck showed notable adventitial inflammation of the vertebral arteries as well as irregularity and diffuse narrowing, with the left side involvement greater than the right side. There was focal area of superimposed short segment stenosis at C2-C3 with poststenotic dilatation on the left side. Myositis of the neck muscles was observed on the left side (Figure 4).

The MRA of his chest, abdomen, and pelvis were normal. MRA of forearm was not obtained. Due to the finding of chronic, stenotic vertebral artery vasculitis, he was admitted for additional workup and aggressive inpatient treatment.

During admission, his additional workup included consultation with neuroradiology who recommended CT Angiography of head and neck, which were obtained to further delineate his vasculitis especially as previous MRA did not include intracranial vessels. Bilateral vertebral artery vasculitis was confirmed by CT Angiography. Left vertebral artery showed stenosis at C2-C3 with poststenotic dilatation from C2 to basal skull. Due to the periosteal reaction of his right forearm, pediatric oncology and orthopedic oncology were consulted to evaluate the bony changes. The periosteal thickening was considered to be most likely a reaction from local myositis; therefore bone and muscle biopsies were deferred. Of note, the patient already did have a bone marrow biopsy done at the outside hospital, which was negative 2 months prior to his initial visit to pediatric rheumatology.

**Figure 5:**
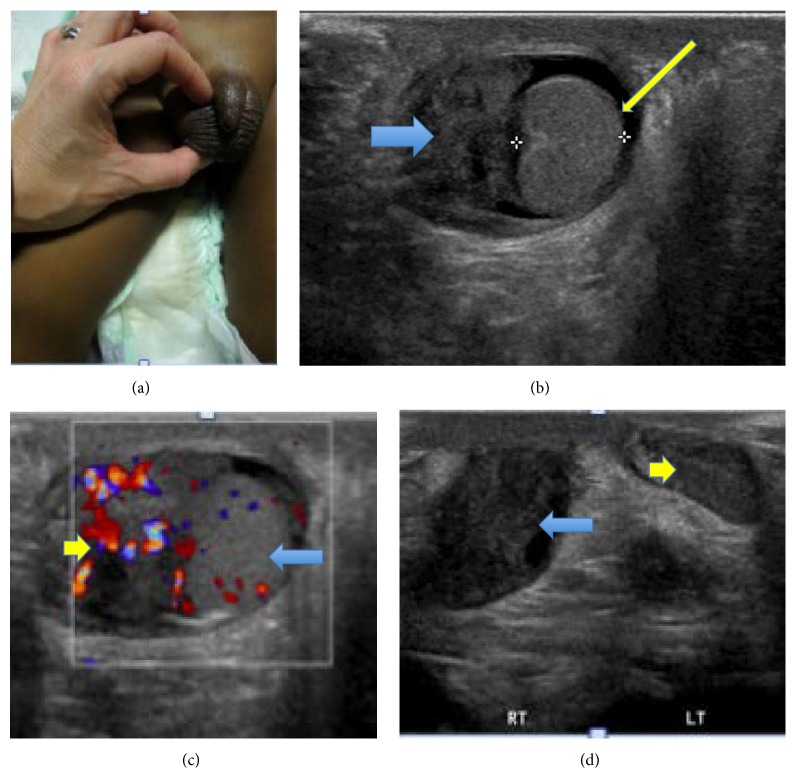
Physical exam and imaging of right testis at diagnosis. (a) Enlarged right testis. (b) Baseline ultrasound of the scrotum demonstrated enlarged right testis (thin yellow arrow) and epididymis (thick blue arrow). (c) Testicular Doppler showing hyperemic right testis (long blue arrow) and epididymis (short yellow arrow). (d) Right side testicle (long blue arrow) was estimated to be approximately twice the volume of the left (short yellow arrow).

Pediatric urology was consulted due to his testicular enlargement and pain. Alfa fetal protein (AFP) and beta-human chorionic gonadotropin (HCG) tumor markers were obtained for concerns of scrotal enlargement, which were negative. Scrotal ultrasound (US) with Doppler (Figure 5) showed increased size of right epididymis and testis with increased vascularity, suggestive of inflammation; therefore testicular biopsy was deferred.

**Figure 6:**
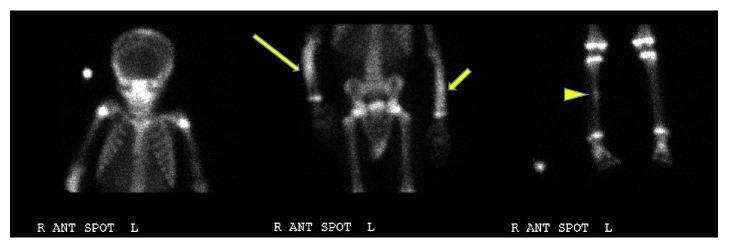
Bone scan at diagnosis. Bone scan: multifocal areas of uptake in the bilateral forearms most pronounced in the right ulna and radius (thin, long yellow arrow) as well as the left ulna and radius (thin short yellow arrow) and right proximal tibia (arrowhead). Gallium scan (not shown) obtained at this time demonstrated subtle increased uptake corresponding to the areas of increased activity on the bone scan, but the activity was less intense than the bone scan, supporting this to be inflammatory process. There was also soft tissue uptake in the right forearm.

Bone scan was done to further evaluate his periostitis. There was diffuse increased uptake in the right forearm. Interestingly, in addition to the expected areas of increased activity, namely, the right forearm, few additional clinically occult areas also showed increased uptake. There was also diffuse uptake in the left forearm similar in the extent and activity and focal uptake was noted in right mid tibia (Figure 6).

**Figure 7:**
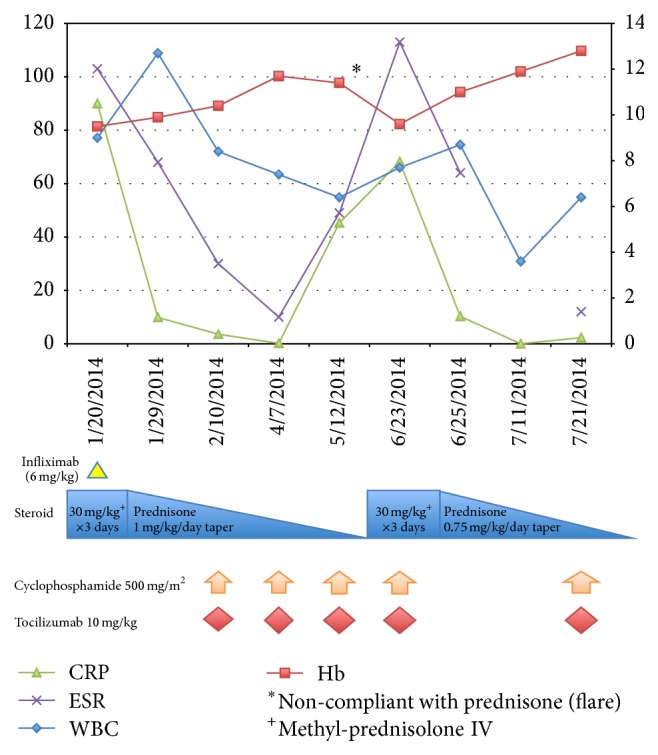
Summary of treatment course and laboratory parameters.

**Figure 8:**
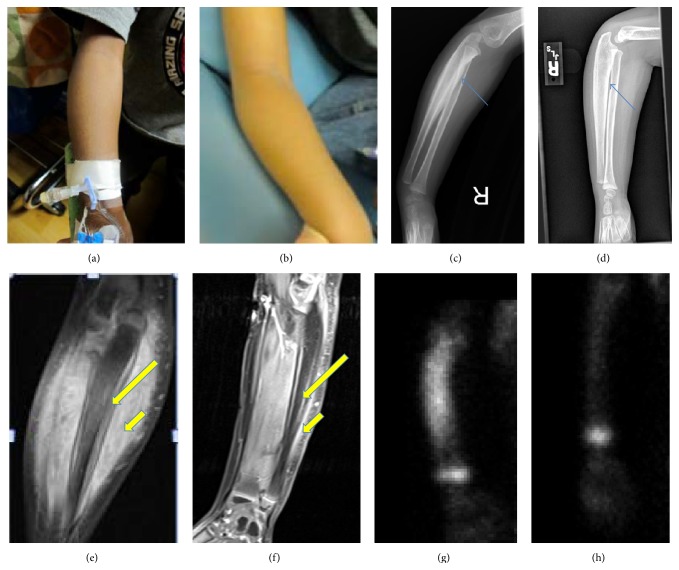
Comparison of right arm before and after treatment with exam and images. (a) Right arm before treatment. (b) Right arm after treatment showing resolution of right forearm hypertrophy. (c) Right arm X-ray before treatment. (d) Right arm X-ray after treatment: follow-up radiographs revealed near total resolution of the periosteal reaction (cf. thin blue arrows). (e) Right arm MRI before treatment. (f) Right arm MRI after treatment: follow-up MRI of the right forearm revealed resolution of periostitis (cf. long yellow arrows) and myositis (cf. short yellow arrows). (g) Right arm bone scan before treatment. (h) Right arm bone scan after treatment: follow-up revealed total resolution of the abnormal uptake in the forearm.

**Figure 9:**
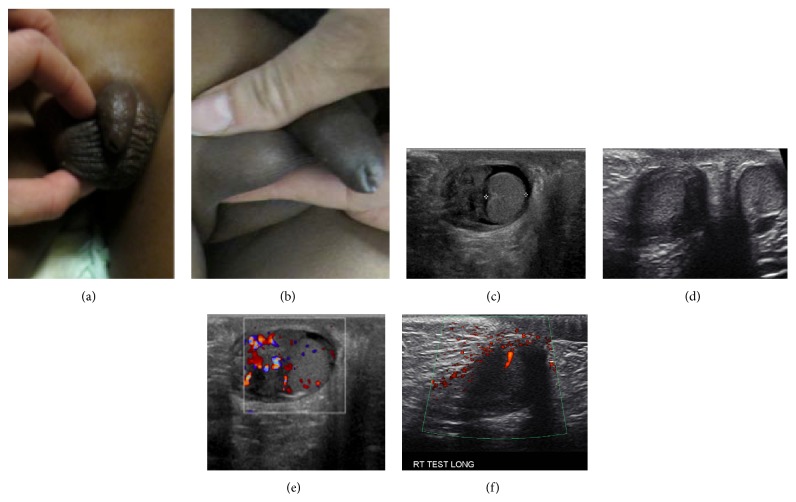
Comparison of right testes before and after treatment with exam and images. (a) Enlarged right testis before treatment, (b) normal sized right testis after treatment. Both testes (not shown) on follow-up exam were equal in size and volume. (c) Right testicular US before treatment. (d) Right testicular US after treatment: follow-up scrotal ultrasound demonstrated normal appearing right testis and epididymis with equal volume and size compared to left. (e) Right testicular Doppler before treatment shows hyperemia. (f) Right testicular Doppler after treatment shows normal Doppler flow.

**Figure 10:**
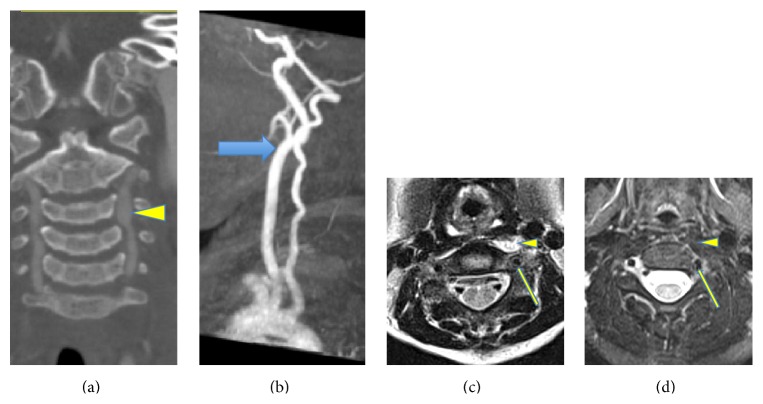
Neck imaging before and after treatment. (a) CTA coronal reformats demonstrating bilateral vertebral arteries prior to treatment. There is focal stenosis at C2-3 with poststenotic dilatation on the left side (arrowhead); the caliber of the right vertebral artery is maintained. (b) Left vertebral sagittal MRA after treatment demonstrating resolution of stenosis and poststenotic dilatation (thick blue arrow). (c) Axial MRI of cervical spine through C2 before treatment demonstrated marked adventitial thickening of bilateral vertebral arteries and focal stenosis at C2-C3 on the left side (arrow) and cervical myositis (arrowhead). (d) Posttreatment images demonstrated improvement in the adventitial thickening of bilateral vertebral arteries with improvement in the caliber of the artery at C2-3 (arrow). Note that there is complete resolution of muscular edema (arrowhead).

**Figure 11:**
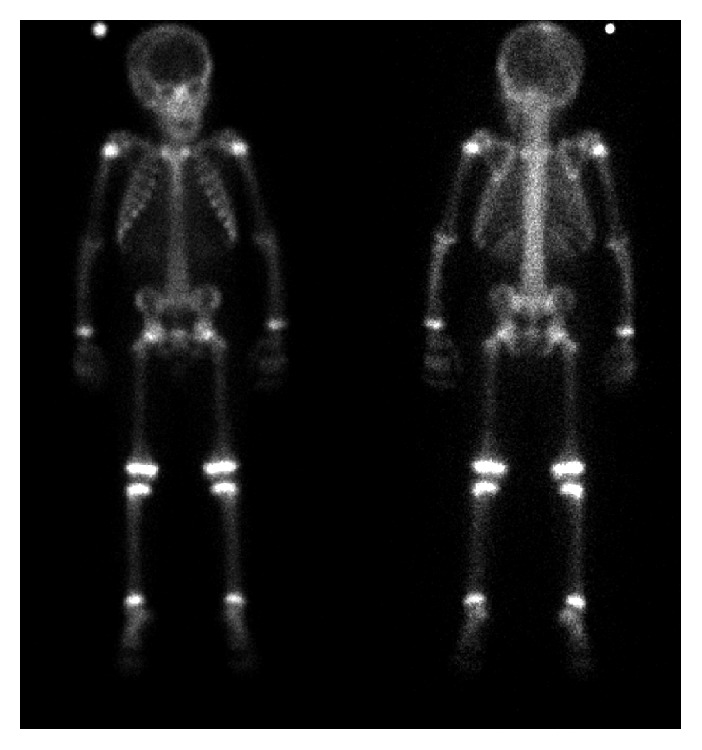
Posttreatment bone scan. Follow-up bone scan after the completion of the treatment demonstrated no focal activity, consistent with complete response to the therapy.

Initially, the patient received scheduled intravenous ketorolac (0.5 mg/kg/dose every 6 hours) for his arthritis and myositis symptoms, which resulted in clinical improvement of his scrotal swelling as well as arm and neck pain. Additionally, his ESR and CRP also improved. Due to his extensive and chronic nature of his bilateral vertebral artery vasculitis, as well as the concern regarding the extracranial location of the vasculitis, he was initially treated with pulse methylprednisolone (30 mg/kg/dose daily × 3) and received one dose of infliximab (6 mg/kg) while inpatient. Subsequently he received a total of 7 monthly doses of cyclophosphamide (500 mg/m^2^) for his vasculitis and monthly tocilizumab for his vasculitis and arthritis with high dose oral steroid taper starting at 1 mg/kg daily (Figure 7). This aggressive, combined approach was decided due to the location and bilaterality of the vertebral artery vasculitis, as well as the chronicity of his symptoms and the presence of stenosis in order to circumvent life-threatening complications such as rupture or worsening stenosis in this critical area with limited ability to form collaterals. This treatment resulted in clinical improvement of his myositis, arthritis, ostitis, and epididymoorchitis as well as serologic improvement in his inflammatory markers and radiographic improvement in his vasculitis. Physical examination and follow-up imaging at 7 months after initiation of treatment showed resolution of his right forearm hypertrophy and near total resolution of periosteal reaction and myositis (Figure 8). Follow-up testicular exam and scrotal Doppler US showed normal appearing right testis and epididymis as well as normal vascularity of the right testis (Figure 9). Most importantly the focal residual irregularity of the wall of the vertebral arteries and adventitial thickening had markedly improved (Figure 10). Furthermore, on bone scan there was total response to the treatment with no abnormal residual activity (Figure 11).

## 3. Discussion

PAN is a rare systemic necrotizing arteritis involving small- and medium-sized muscular arteries with multisystem involvement making it challenging to diagnose. In case of being untreated or delayed treatment, PAN can be associated with significant morbidity and mortality. Childhood PAN has also been referred to as infantile KD or infantile PAN which are both on the spectrum of necrotizing vasculitis that can involve the coronary arteries, but the use of the term infantile (which connotes < 1 year) is too restrictive and in our case this patient was older. The patient's coronary artery dilation resolved after one dose of IVIG and due to the latter development of more extensive symptoms of vertebral artery vasculitis, epididymitis, periostitis, and myositis, which are not typical of KD, we believe that his coronary artery dilation was likely part of his childhood PAN. Of note, a childhood onset of vasculopathy overlapping polyarteritis nodosa caused by a single gene defect in CECR1 (Cat Eye Syndrome Chromosome Region Candidate 1) resulting in deficiency of adenosine deaminase 2 has also been reported in families with autosomal recessive inheritance pattern [12]. Our patient was not tested for this condition at this time given lack of familial involvement but may be warranted in the future.

About 50% of children with newly diagnosed PAN present with musculoskeletal symptoms including joint, muscle, or limb pain [2]. Therefore, evaluation of periostitis, a more challenging diagnosis to make, is important in evaluating the extent of disease in addition to arthritis and myositis. Cases of periostitis and periosteal reactions in patients with PAN have been reported [9, 10, 13–15], but rarely in pediatrics. This finding may have clinically silent areas that can be detected by bone scan imaging modalities.

It has been stated that most periosteal reactions are found in long bones especially in the lower limb [13, 14]. In our case, his long bone involvement was his right ulna and radius as well as his right tibia and left radius and ulna, but these latter areas were clinically silent and notable on bone scan imaging only. Localized vasculitis is said to be responsible for local production of bone growth factors associated with hypoxia, which could participate in the pathogenesis of periostitis [15]. In our case we had initially thought it was a secondary inflammation due to myositis that was localized in the same area, but it is hard to evaluate which came first. The patients right arm hypertrophy was not noted by the family or other treating physicians, possibly since it did not affect the patient's range of motion.

In several cases of PAN bone scan has been utilized for evaluation of extremity pain [9, 10, 13], which can be very useful in pediatric patients who may have difficultly in communicating pain level and location. In our case the muscle and bone biopsy were deferred and the patient met criteria of PAN with imaging studies and his other presenting symptoms. Obtaining periosteal histology may show evidence of necrotizing arteritis, although often the changes seen are nonspecific such as bone reabsorption and formation [14]. Cases with periostitis may or may not involve localized overlying cutaneous PAN lesions [14] which were absent in our patient. Bone scan may be a useful first-line examination in detecting subclinical and extra-articular involvement of inflammatory bone processes [9, 10, 13], especially in the pediatric population, as it is a low invasive test.

CNS involvement has been reported in up to 22% of cases of polyarteritis nodosa in the pediatric population, presenting with encephalopathy, focal deficits, stroke, and seizures [2–5]. The most common neuroradiological finding is focal ischemic areas, followed by intracranial hemorrhage with narrowing or occlusion of intracranial arteries on angiography [16]. There have been no reports on vertebral artery occlusion, which would be more common in large vascular arteritis including primary CNS angiitis, Takayasu Arteritis, and Giant Cell Arteritis, however our patient did not meet EULAR criteria [7]. Our patient did not present with neurologic symptoms. In patients with rheumatic disease, neck pain is often associated with arthritis, but in our case, we were able to diagnose radiographically cervical myositis and vasculitis as a contributing cause of neck pain. Due to the presence of chronic, bilateral vertebral artery vasculitis, with delayed treatment and focal stenosis, we decided to treat our patient more aggressively due to the potential deleterious effects of extracranial vascular injuries including further progression, stenosis, stroke, aneurysm formation, rupture, or hemorrhage [4, 5, 16, 17]. We believe that our treatment plan prevented intracranial progression and embolic/ischemic stroke. Collaterals were not observed in our patient, as the stenosis was less than 70%. Our initial aggressive approach included treatment with combination of high dose pulse corticosteroids, cyclophosphamide, and tocilizumab due to his vasculitis and arthritis. He did receive one initial dose of infliximab, which was available on the inpatient formulary for his arthritis and vasculitis.

Tocilizumab use has been described mainly in juvenile idiopathic arthritis, but in limited case reports, usage in Giant Cell Arteritis and Takayasu's Arteritis [11, 18] has shown benefit for treatment of vasculitis. The use of tocilizumab in PAN has not been approved by the United States Food and Drug Administration (FDA), but the blockage of IL-6 with tocilizumab has been used effectively to treat other forms of vasculitis [19, 20]. Furthermore in our case we had concomitant use of cyclophosphamide for his extracranial involvement. In spite of this combination of immunosuppressants, our patient has been infection-free during the 7 months of concomitant treatment and has not had common or severe adverse effects seen in tocilizumab such as upper respiratory infection, pharyngitis, diarrhea, increase in liver enzymes, hyperlipidemia, and neutropenia [18]. This concomitant use of tocilizumab, cyclophosphamide, and steroids has improved the systemic manifestations of vasculitis, myositis, arthritis, periostitis, and epididymoorchitis in our patient based on clinical exam, serologic markers, and follow-up imaging at 7 months. Future studies are recommended for the use of tocilizumab and combination therapy in PAN. For our patient, follow-up imaging is planned to assess ongoing response to treatment.

## 4. Conclusion 

Periostitis can be an initial presentation of PAN in the pediatric population. It may be clinically silent in some areas but could be helpful in differentiating PAN from other vasculitis. Bone scintigraphy may be utilized as a noninvasive modality to evaluate and monitor inflammation and response to treatment for periostitis. To our knowledge, we have also presented the first case of PAN presenting with vertebral artery dilatation and aneurysm development that has responded to combination treatment with cyclophosphamide, glucocorticoids, and tocilizumab. Therapeutic trials are needed to determine the efficacy and use of tocilizumab in the treatment of PAN and in combination therapy.

## Abbreviations

PAN: Polyarteritis nodosa
CNS: Central nervous system
KD: Kawasaki disease
MRI/A: Magnetic Resonance Imaging/Angiography (MRI/A)
EULAR: The European League Against Rheumatism
PReS: Pediatric Rheumatology European Society
ACR: American College of Rheumatology
IV: Intravenous
CRP: C-reactive protein
ESR: Erythrocyte Sedimentation Rate
AST: Aspartate aminotransferase
ALT: Alanine aminotransferase
WBC: White blood cell
RPR: Rapid plasma reagin
CMV: * Cytomegalovirus*
HIV: Human Immunodeficiency Virus
PCR: Polymerase chain reaction
AFP: Alfa fetal protein
HCG: Human chorionic gonadotropin
US: Ultrasound
FDA: Food and Drug Administration.
